# Host cell responses to *Candida albicans* biofilm-derived extracellular vesicles

**DOI:** 10.3389/fcimb.2024.1499461

**Published:** 2025-01-14

**Authors:** Kamila Kulig, Ewelina Wronowska, Magdalena Juszczak, Marcin Zawrotniak, Justyna Karkowska-Kuleta, Maria Rapala-Kozik

**Affiliations:** ^1^ Department of Comparative Biochemistry and Bioanalytics, Faculty of Biochemistry, Biophysics and Biotechnology, Jagiellonian University, Kraków, Poland; ^2^ Doctoral School of Exact and Natural Sciences, Faculty of Biochemistry, Biophysics and Biotechnology, Jagiellonian University, Kraków, Poland

**Keywords:** extracellular vesicles, *Candida albicans*, biofilm, host immune response, pathogenic fungi, candidiasis

## Abstract

*Candida albicans* is a prevalent fungal pathogen responsible for infections in humans. As described recently, nanometer-sized extracellular vesicles (EVs) produced by *C. albicans* play a crucial role in the pathogenesis of infection by facilitating host inflammatory responses and intercellular communication. This study investigates the functional properties of EVs released by biofilms formed by two *C. albicans* strains—3147 (ATCC 10231) and SC5314—in eliciting host responses. We demonstrate the capability of *C. albicans* EVs to trigger reactions in human epithelial and immune cells. The involvement of EVs in pathogenesis was evidenced from the initial stages of infection, specifically in adherence to epithelial cells. We further established the capacity of these EVs to induce cytokine production in the epithelial A549 cell line, THP-1 macrophage-like cells, and blood-derived monocytes differentiated into macrophages. Internalization of EVs by THP-1 macrophage-like cells was confirmed, identifying macropinocytosis and phagocytosis as the most probable mechanisms, as demonstrated using various inhibitors that target potential vesicle uptake pathways in human cells. Additionally, *C. albicans* EVs and their cargo were identified as chemoattractants for blood-derived neutrophils. After verification of the *in vivo* effect of biofilm-derived EVs on the host, using *Galleria mellonella* larvae as an alternative model, it was demonstrated that vesicles from *C. albicans* SC5314 increased mortality in the injected larvae. In conclusion, for both types of EVs a predominantly pro-inflammatory effect on host was observed, highlighting their significant role in the inflammatory response during *C. albicans* infection.

## 1 Introduction

Host cells deploy variety mechanisms to respond to and defend against microbial infections, caused by both bacteria and fungi. Epithelial cells, situated at the forefront of this defensive line, play a pivotal role in the initial stages of infection development. Their response influences subsequent reaction from the other cells, like immune cells. The signaling molecules released by host cells facilitate intercellular communication orchestrating the overall inflammatory response. Additionally, environmental factors and diseases can profoundly affect the organism’s homeostatic balance.

The fungal commensal *Candida albicans* becomes pathogenic when host homeostasis is disrupted, which is associated with impairment of host immunity, causing mild superficial and serious systemic candidiases. The virulence mechanisms performed by *Candida* yeasts are based on the release of hydrolytic enzymes, quorum sensing molecules that facilitate microorganisms’ communication, presence of adhesins on the yeast cell surface, the change of morphological forms and formation of biofilm structures. Another mechanism contributing to their virulence is the production of extracellular vesicles (EVs), which are nanosized structures with lipid bilayer and various cargo molecules. These vesicles play a crucial role in the pathogenicity of yeasts, mediating different aspects of host-pathogen interactions; nonetheless, they may also have a protective effect against *C. albicans* infection as they contain numerous proteins with antigenic properties (Gil-Bona et al., 2015; Vargas et al., 2015, 2020; Brandt et al., 2024; Honorato et al., 2024).

The production of EVs by yeasts from the *Candida* genus has been well-documented in numerous studies, for both *C. albicans* (Gil-Bona et al., 2015; Vargas et al., 2015, 2020; Zarnowski et al., 2018; Zamith-Miranda et al., 2021; Martinez-López et al., 2022) and non-*albicans Candida* species such as *C. auris* (Zamith-Miranda et al., 2021), *C. haemulonii* (Oliveira et al., 2023), *C. tropicalis, C. parapsilosis* (Karkowska-Kuleta et al., 2020; Kulig et al., 2022), and *Nakaseomyces glabratus* (the latter was previously included in the genus *Candida* and known as *C. glabrata*) (Karkowska-Kuleta et al., 2020; Kulig et al., 2022; Kidd et al., 2023). EVs act as pivotal carriers of proteins, lipids, quorum sensing molecules and other compounds critical for host-pathogen interactions. They play a dual role, not only triggering the host’s immune response, but also facilitating communication between microorganisms. This communication is crucial for regulating growth and morphological changes that are essential for the progression of infection. By mediating these interactions, EVs contribute significantly to the complexity of microbial pathogenicity and the host defense mechanisms (Kullberg and Arendrup, 2015; da Silva Dantas et al., 2016; Nimrichter et al., 2016; Woith et al., 2019; Rizzo et al., 2020; Chow et al., 2021; Liebana-Jordan et al., 2021; Zamith-Miranda et al., 2021; Zhou et al., 2021; Bitencourt et al., 2022; Lopes and Lionakis, 2022; Patel, 2022; Stotz et al., 2022). During the development of *C. albicans* infection, fungal cells have been proven to invade epithelial cells, especially after transformation to the more invasive hyphal form (Maza et al., 2017). Moreover, *C. albicans* EVs have been shown to regulate the differentiation from yeast to hyphal cells during formation of the biofilm by *C. albicans* (Honorato et al., 2022).

The ability to release numerous cytokines, such as IL-1β, IL-6, IL-8, IL-10 or TNF-α, by host cells in the response to microbial virulence factors has been reported (Eckmann et al., 1993; Krueger et al., 1991; Taylor et al., 1999; Waterhouse and Stadnyk, 1999; Hyun et al., 2015; Peng et al., 2015). Although TNF-α is known to be produced mainly by macrophages (Jang et al., 2021), epithelial cells were observed to release this cytokine during infection or hypoxia (Taylor et al., 1999; Roulis et al., 2011). Further, the production of IL-10 influences the integrity of epithelial cells, and the lack of this molecule can decrease this process leading to facilitating adhesion and invasion of pathogens (Abreu, 2010; Hyun et al., 2015). The immune cells while fighting with microbial infections can generate reactive oxygen species (ROS), produce cytokines, and activate phagocytosis or extracellular trap formation (Naglik, 2014; Netea et al., 2015; Herb and Schramm, 2021). As the content of EVs, comprised of numerous molecules identified as virulence factors, was reported to induce various types of the host responses, the potential of fungal EVs to elicit such functional role and their participation in these processes is an intriguing topic for investigation. In the current study, we demonstrated the involvement of *C. albicans* EVs derived from biofilm-forming fungal cells of two *C. albicans* strains – 3147 and SC5314 – in eliciting host immune response by affecting epithelial cells, neutrophils and macrophages. We focused on (i) the engagement of EVs in the adhesion process of fungal cells to epithelium, (ii) the production of cytokines, mainly IL-1β, TNF-α, IL-8 and IL-10, by epithelial or macrophage cells, (iii) the involvement of *C. albicans* EVs in chemotaxis of neutrophils and (iv) the ability to internalize these fungal structures by macrophages. Furthermore, we indicated the possible mechanisms of EVs uptake activated by human macrophages. In addition, the survival of *Galleria mellonella* larvae was monitored after the injection of *C. albicans* EVs to determine the *in vivo* effect of fungal structures.

## 2 Materials and methods

### 2.1 Fungal strains, culture conditions and EVs isolation

The pre-culture of *Candida albicans* strains 3147 (ATCC^®^ 10231) and SC5314 yeasts (ATCC, Manassas, VA, USA) was performed for 18 h at 30°C in an orbital shaker MaxQ 6000 (Thermo Fisher Scientific, Waltham, MA, USA) with rotary speed of 170 rpm in 20 ml of YPD medium (1% yeast extract, 2% soybean peptone, and 2% glucose, Sigma, St. Louis, MO, USA). Then, 1 × 10^8^ C*. albicans* cells from the pre-culture were inoculated into 100 ml of RPMI 1640 medium (Biowest, Nuaille, France) and cultured for 48 h (with medium exchange after 24 h) at 37°C in sterile roller bottles (Corning Inc., New York, NY, USA) with a roller rack speed of 3 rpm for biofilm culture, as described previously (Zarnowski et al., 2018; Karkowska-Kuleta et al., 2023).

To isolate EVs, the centrifugation was performed at 4000 × *g* for 15 min at 4°C on combined samples of culture medium collected every 24 h from the biofilm culture. This step was repeated twice, discarding the cell pellet after each centrifugation. Subsequently, the complete Protease Inhibitor Cocktail (Roche, Basel, Switzerland) was added to the supernatants, and the concentration of the supernatants was achieved using an Amicon Ultra-15 Centrifugal Filter Unit with a 100-kDa cutoff (Merck, Darmstadt, Germany). Further centrifugation was conducted for 5 min at 5000 × *g*, followed by discarding of pellet. The concentrated medium was then filtered through an Ultrafree-CL Centrifugal Filter (Merck, Darmstadt, Germany) with a pore size of 0.65 μm, to eliminate cellular debris. The final ultracentrifugation step was carried out for 1 h at 45 000 rpm, corresponding to a relative centrifugal force of 144 000 × *g* (*k* factor 112) at 4°C in polycarbonate thick-wall centrifuge tubes (13 × 64 mm) equipped with 13-mm-diameter Delrin tube adapters, using a fixed-angle type 60 Ti Rotor in an Optima™ LE-80K Ultracentrifuge (Beckman Coulter, Brea, CA, USA).

The collected EVs were suspended in phosphate buffered saline (PBS) filtered through a 0.22 μm filter, in Eppendorf tubes and stored at -80°C for further use.

### 2.2 Characterization of fungal EVs

To determine the protein concentration in the samples containing EVs, the *o*-phthalaldehyde (OPA) assay (Sigma) with the modification of the primary amine groups of proteins was performed, involving measuring the fluorescence intensity of the samples, with excitation at 340 nm and emission at 455 nm as previously described (Karkowska-Kuleta et al., 2020).

The phospholipid concentration was determined using the Phospholipid Assay Kit MAK122 (Sigma), and the carbohydrate concentration was assayed with the Total Carbohydrate Assay Kit MAK104 (Sigma), strictly in accordance with the manufacturer’s instructions, with the absorbance measurements at 570 nm (MAK122) or 490 nm (MAK104). All measurements were performed in three biological replicates for EVs from both fungal strains, with a Synergy H1 microplate reader (BioTek Instruments, Winooski, VT, USA).

### 2.3 Human cell lines and culture conditions

The A549 human lung adenocarcinoma cell line (ATCC^®^ CCL-185™) was cultured in F-12K medium (Kaighn’s modification of Ham’s F-12 medium) (Corning) supplemented with 10% fetal bovine serum (FBS, Gibco) and with 100 U/ml penicillin and 100 mg/ml streptomycin (Biowest).

The BJ fibroblast cell line (ATCC^®^ CRL-2522™) was cultured in Eagle’s Minimum Essential Medium (EMEM) supplemented with 1% sodium pyruvate and 1% nonessential amino acids (each from Biowest), 10% FBS (Gibco) and 100 U/ml penicillin and 100 mg/ml streptomycin (Biowest).

The THP-1 human monocytic cell line (Sigma-Aldrich) was cultured in RPMI 1640 medium (Biowest) supplemented with FBS (Gibco). To induce differentiation of monocytes into adherent macrophage-like cells, phorbol 12-myristate 13-acetate (PMA) was added to the medium at a final concentration of 10 ng/ml along with 100 U/ml penicillin and 100 mg/ml streptomycin (both from Biowest). This treatment was conducted for 48 h, with a medium exchange after the first 24 h.

All cell cultures were maintained at 37°C in an atmosphere containing 5% CO_2_ and 95% humidity.

### 2.4 Isolation of blood-derived neutrophils and monocytes

As described previously (Smolarz et al., 2021), neutrophils and monocytes were isolated from whole-blood samples treated with 5 mM EDTA obtained from healthy donors at the Regional Blood Donation Center (Kraków, Poland). All procedural steps were carried out at room temperature using reagents preheated to 37°C. Initially, the morphotic elements were separated from the plasma by centrifugation at 420 × *g* for 20 min. Subsequently, one-third of the upper plasma phase along with the bottom layer containing leukocytes and erythrocytes, was transferred to a new tube and brought up to a volume of 20 ml with sterile PBS, followed by mixing through tube inversion. The remainder of the sample was discarded. Using the lymphocyte separation medium (Biowest, Nuaille, France) the further separation of the cells was performed by overlaying the sample and centrifuging at 420 × *g* for 30 min. The layer containing peripheral blood mononuclear cells (PBMCs) was collected into a new 50 ml tube for subsequent monocyte isolation, while the lower phase containing granulocytes and erythrocytes was collected for the isolation of neutrophils.

To the collected lower phase, two volumes of 1% polyvinyl alcohol (PVA) was added and the mixture was allowed to stand for 20 min to facilitate the separation of erythrocytes from granulocytes. Then, the upper phase containing granulocytes was transferred into a new tube. The sample was then centrifuged for 5 min at 420 × *g*, and 1 ml of Red Blood Lysis Buffer (Roche, Penzberg, Germany) was added to the resultant cell pellet. After another round of centrifugation, the cells were washed with PBS. The isolated granulocytes were finally resuspended in 1 ml of DMEM medium without phenol red (Biowest, Nuaille, France), and the experiments were conducted immediately following isolation.

To isolate monocytes, the collected layer containing PBMC was adjusted to the volume of 50 ml with cold PBS and centrifuged at 300 × *g* for 10 min at 4°C. This step was repeated and followed by the removal of supernatant. After last discarding of the supernatant, the cell pellet was resuspended in cold 1640 RPMI medium (Biowest). Approximately 4 million PBMCs per well were then seeded onto a 24-well plate in the total volume of 2 ml of RPMI medium (without FBS) and incubated for 3 h at 37°C in the atmosphere of 5% CO_2_ and 95% humidity.

After incubation, cells were washed twice with PBS (pH 7.4) by pipetting up and down to remove non-adherent lymphocytes. The monocytes that adhered to the microplate were then suspended in 2 ml of RPMI 1640 medium supplemented with 10% of FBS and M-CSF (recombinant human macrophage colony-stimulating factor) at a final concentration of 10 ng/ml per well. The monocytes were incubated for 7 days to differentiate them into monocyte-derived macrophages (MDM) with medium exchanges occurring every 2-3 days.

### 2.5 Adhesion assay

The impact of the fungal EVs on the adhesion process of *C. albicans* cells was analysed using epithelial A549 cells and BJ fibroblast cells. Initially, 2 × 10^4^ human cells per well were seeded in F-12K medium or EMEM medium supplemented with 10% of FBS and cultured for 24 h at 37°C to form monolayer of the epithelial or fibroblast cells. Subsequently, the medium was replaced with RPMI 1640 containing 10% of FBS. At this time, fungal EVs were added at a ratio of the host cells to *C. albicans* EVs of 1:10^5^, along with yeast cells at density of 5 × 10^5^ or 1 × 10^6^ cells per ml, for microscopic analysis or colony-forming unit (CFU) counting, respectively, in the total volume of 100 μl of medium. After 3 h of incubation at 37°C, cells were washed twice with sterile PBS. For microscopic analysis, cells were fixed with 4% (w/v) of paraformaldehyde (PFA) solution for 20 min at room temperature, washed twice with sterile PBS and then yeasts were stained with Calcofluor White Stain (Sigma-Aldrich) using stock solution (1 mg/ml), which was diluted a thousand times in PBS and added to the cells for 15 min with protection from light to bind to fungal cell wall. Following this staining step, cells were washed with sterile PBS, and 100 µl of fresh PBS was added for microscopic analysis performed with the use of an Olympus IX73 microscope (Olympus, Tokyo, Japan), Hamamatsu Orca Spark camera (Hamamatsu, Hamamatsu City, Japan), and a UPLXAPO60XO lens (Olympus). The images were analyzed using Olympus CellSens Dimension 3.1 software. The number of fungal cells were manually counted from eight microscopic images for each sample, considering four different microscopic fields of view. For the CFU counting, the cells were washed with PBS, then scraped from the plate in the total volume of 50 μl. Serial dilutions were performed in sterile PBS with vigorous pipetting and vortexing the samples before they were spread on YPD agar plates. The plates were further incubated for 48 h at 30°C for the growth of *C. albicans* colonies. The colonies were manually counted and the results were presented as colony forming units per ml (CFU/ml).

### 2.6 Stimulation of human cells by *C. albicans* EVs

For the stimulation of the A549 cells, 24 h after seeding of 2 × 10^5^ cells per well in a 24-well microplate, the growth medium was replaced with fresh F-12K medium containing EVs, at a cell to EVs ratio of 1:10^5^. The cells were then incubated for 24 h at 37°C. After differentiating of 5 × 10^5^ THP-1 cells per well in a 24-well microplate into adherent macrophage-like cells using 10 ng/ml PMA for 48 h as previously described, the growth medium was exchanged with fresh medium devoid of PMA, but with 5% FBS, and cells were allowed to rest for 3 h. Subsequently, medium was replaced with fresh RPMI 1640 medium and obtained macrophage-like cells were incubated with EVs, at a cell to EVs ratio of 1:10^5^ for 24 h at 37°C. Supernatants from above the cells were collected for further analysis of cytokine secretion, and genetic material was isolated from the cells for the analysis of gene expression.

### 2.7 Gene expression analysis

The isolation of RNA was performed with ReliaPrep™ RNA Cell Miniprep System (Promega) in accordance with manufacturer’s instructions, and the obtained RNA samples were stored at -80°C. To synthetize cDNA, Moloney murine leukemia virus reverse transcriptase (M-MLV RT, Promega) was used in the following procedure including the incubation for 5 min at 70°C of the reaction mixture containing 2 μg of RNA with added 2 μl of dT18 primers in the concentration of 100 μM, and the volume brought to 15 μl with RNase-free water. This was followed by placing sample on the ice and adding 5 μl of 5× concentrated transcriptase buffer, 1.25 μl of the dNTP mixture (Thermo Fisher Scientific, 10 mM), 2.75 μl of RNase-free water, and 1 μl of M-MLV transcriptase (Promega, 200 U). Then the sample was mixed, centrifuged, and incubated for 60 min at 42°C and 10 min at 70°C in the thermocycler. The obtained cDNA samples were stored at -20°C, and for further use in the quantitative real-time qPCR (RT-qPCR) analysis, were diluted 4 times in RNase-free water.

The gene expression analysis with RT-qPCR was performed in 96-well reaction plates (MicroAmp ™ Fast Optical 96-well reaction plate) by adding 2 μl of diluted cDNA and 8 μl of a reaction mixture (containing 2.6 μl of RNase-free water, 0.2 μl forward primer and 0.2 μl reverse primer, both at the concentration of 10 μM, and 5 μl of SYBR KAPA (Universal Mix KAPA Biosystems, KAPA SYBR^®^ FAST Master Mix (2X) ABI Prism ™)). The reaction and quantitative analysis were performed using a thermal cycler QuantStudio 3 and QuantStudio Design & Analysis software v1.5.2 (Applied Biosystems). As a reference gene, the GAPDH gene was used. The sequences of used primers are listed in **Table 1**.

**Table 1:** Primer description for the mRNA expression analysis.

Gene coding	FORWARD (Sequence 5’ ➔ 3’)	REVERSE (Sequence 5’ ➔ 3’)	Reference
GAPDH	GGGAAGCTTGTCATCAATGG	CATCGCCCCACTTGATTTTG	(Kambas et al., 2011)
IL-8	CACCGGAAGGAACCATCTCACT	CACCGGAAGGAACCATCTCACT	(Karkowska-Kuleta et al., 2018)
IL-1β	CTTTGAAGCTGATGGCCCTAAA	AGTGGTGGTCGGAGATTCGT	(Oudhoff et al., 2010)
IL-6	GGCACTGGCAGAAAACAACC	GGCAAGTCTCCTCATTGAATCC	(Oudhoff et al., 2010)
TNF-α	TCCTCCAGACACCCTCAACC	AGGCCCCAGTTTGAATTCTT	(Karkowska-Kuleta et al., 2018)
IL-10	TGAGAACCAAGACCCAGACA	TGAGAACCAAGACCCAGACA	(Karkowska-Kuleta et al., 2018)

### 2.8 Cytokine production assay

The analysis of cytokine production following stimulation of human cells with EVs, as described above, was performed using collected supernatants. As the negative or positive controls, the supernatants collected from unstimulated cells or those stimulated with LPS (100 ng/ml; Sigma-Aldrich) were used, respectively. To determine the level of selected cytokines − IL-1α, IL-1β, IL-8, tumor necrosis factor α (TNF-α) or IL-10 − released by human cells, quantitative tests were performed using ELISA MAX™ Deluxe Set Human IL-1α (BioLegend), Human IL-1β ELISA Set II, Human IL-8 ELISA Set, Human TNF ELISA Set and Human IL-10 ELISA Set kits (BD OptEIA™), respectively. These analyses were carried out strictly following the manufacturer’s instructions.

### 2.9 Chemotaxis assay

To analyze the impact of *C. albicans* EVs on the chemotaxis of neutrophils, the 24-well chemotaxis chamber technique was employed using Transwell^®^-Clear inserts (Corning, NY, USA) with 3 µm pore size. The number of 1 × 10^6^ neutrophils per insert were seeded in DMEM medium. The inserts were placed in a 24-well chamber, and DMEM medium, supplemented with fungal EVs, at a cell to EVs ratio equal 1:10^5^, was added to the wells. PBS or N-formylmethionyl-leucyl-phenylalanine (fMLP), at a final concentration of 10 μM served as negative and positive controls, respectively. The incubation of the samples was performed for 3 h at 37°C in a 5% CO_2_ atmosphere. Post-incubation, microscopic analysis was conducted to quantify the number of human cells labelled with 1 μM CellTracker Red CMTPX Dye solution (Thermo Fisher Scientific) that migrated to the lower compartment of the chamber with the use of an Olympus IX73 microscope. The images were analyzed using Olympus CellSens Dimension 3.1 software.

### 2.10 Labeling of *C. albicans* EVs with Vybrant DiI Cell-Labelling dye

To prepare fluorescently labeled EVs, 4 × 10^10^ EVs and 2 μl of Vybrant DiI Cell-Labelling solution (Thermo Fisher Scientific) were mixed in 100 μl of PBS, pH 7.4 (Biowest), and incubated for 30 min at room temperature in the dark to allow the dye to bind to the EVs (Mehanny et al., 2020). Then, the sample was diluted with PBS, and the unbound DiI dye was separated from stained EVs by size exclusion chromatography, using qEVoriginal/70 nm columns (IZON Science, Christchurch, New Zealand) (Karkowska-Kuleta et al., 2023), according to the manufacturer’s instructions. The fractions containing EVs were collected, combined and concentrated ten-fold using 10 kDa-centrifugal filter units (Merck). A mock chromatographic separation was also completed with a buffer without EVs, containing only DiI dye, to prepare control samples.

### 2.11 Uptake of EVs

The number of 5 × 10^4^ THP-1 cells were seeded onto 96-well glass-like microplate (Cellvis, Sunnyvale, CA, USA) and differentiated into macrophage-like cells. EVs labeled with DiI dye, at a cell to EVs ratio of 1:10^5^, were added for 24 h incubation at 37°C in RPMI 1640 medium, and then the cells were washed with 200 μl of PBS. To stain cell nuclei, the cells were incubated for 5 min with a solution of 1.2 μg of Hoechst 33342 (Thermo Fisher Scientific) in 100 μl of PBS. The microscopic analysis of cells and EVs was performed using Leica Stellaris confocal microscope (Leica, Wetzlar, Germany). The images were analyzed using LASX Office 1.4.7 software. As representative results the images showing the plane of the cell nucleus were presented. To demonstrate the localization of EVs in the cell as a rotating visualization, a sequence of images showing different planes of view of the each sample was taken together (**Supplementary Material**).

### 2.12 Identification of the mechanism of EVs uptake by THP-1 macrophages

In the wells of 12-well microplate, 1 × 10^6^ THP-1 macrophage-like cells were incubated for 30 min at 37°C in 1 ml of RPMI 1640 medium without phenol red and without FBS, with added inhibitors – cytochalasin D and wortmannin at a final concentration of 0.5 µM each, chlorpromazine at 5 µM, nystatin at 10 µg/ml, and dynasore at 40 µM – that inhibit actin polymerization, phosphoinositide 3-kinase, clathrin, caveolin, and dynamin activity, respectively (da Costa Gonçalves et al., 2021). Following treatment, the cells were washed twice with 500 µl of fresh RPMI 1640 medium without phenol red and without FBS, then 900 µl of fresh RPMI 1640 medium without phenol red and without FBS, re-supplemented with reversable inhibitors was added, and right after, EVs fluorescently labelled with DiI dye were added to the wells at the cell to EVs ratio of 1:10^5^ in the volume of 100 µl in the same medium. Then cells were incubated for an additional 6 h at 37°C to allow for EVs uptake. After the incubation period, the cells were washed twice with 500 μl of PBS to remove any unbound EVs. Cells incubated with EVs only, without inhibitors at any stage, served as a control for 100% uptake. Next, the adherent cells were collected using trypsinization and fixed with a 4% (w/v) of paraformaldehyde solution. The fixed cells were analyzed using flow cytometry (LSR Fortressa, BD, USA) to assess the uptake of fluorescently labeled EVs.

### 2.13 The survival of *Galleria mellonella* larvae

The evaluation of *in vivo* toxicity of fungal EVs was performed with the use of *Galleria mellonella* moth larvae, collected in separate groups, each of ten randomly selected individuals. With the use of 10 µl Hamilton syringe (Merck), samples contained 1 × 10^8^ fungal EVs of each tested *C. albicans* strain in 10 µl of sterile PBS buffer, pH 7.5 (Biowest), were given to the left proleg of each larva. The injection of 10 µl of sterile PBS buffer served as a control. Larval survival was then monitored for the next 6 days of incubation at 37°C. The mortality of the larvae was verified based on their dark color and lack of movement after stimulation by touch. Six biological replicates were performed for each group.

### 2.14 Statistical analysis

Data are presented as the mean ± standard deviation (SD). To analyze the statistical significance, an unpaired t-test was performed using GraphPad Prism software version 10.0.3 (GraphPad Software, La Jolla, CA, USA). The statistical significance levels were denoted as follows: * for *p* < 0.05, ** for *p* < 0.01, ***for *p* < 0.001, **** for *p* < 0.0001, and ns for not significant.

## 3 Results

The fungal EVs of two *C. albicans* strains – 3147 and SC5314 – were obtained from supernatants collected after 48-h culture of yeasts forming biofilm structure in roller bottles in RPMI 1640 medium, simulating flow culture conditions as described previously (Zarnowski et al., 2018; Kulig et al., 2022; Karkowska-Kuleta et al., 2023). These are two widely researched reference *C. albicans* strains that differ in their virulence and ability to form hyphae and biofilm (Glazier et al., 2023), and previous studies have also characterized EVs produced by both yeast-like cells and hyphal forms of these strains indicating their important role in regulating fungal morphology, biofilm formation and interaction with the host (Honorato et al., 2022; Martinez-López et al., 2022; Karkowska-Kuleta et al., 2023). The characterization of the size and concentration of EVs in obtained preparations, performed using TEM visualization and NTA analysis, has been presented in our recent research (Karkowska-Kuleta et al., 2023), while the global composition of EVs was described herein in terms of protein, phospholipid and carbohydrate content, and included in **Table 2**. The average protein content of EVs from *C. albicans* strain 3147 was slightly lower than that of EVs from *C. albicans* strain SC5314. The phospholipid content for the same number of EVs was almost three times higher for *C. albicans* strain 3147 than for SC5314. The last component examined were carbohydrates, and their content was at comparable levels for both types of EVs.

**Table 2:** Characteristic of protein, lipid, and carbohydrate content and the sizes of EVs produced by cells forming biofilm structure of *C. albicans* strains 3147 and SC5314.

	Protein content (μg per 1x10^10^ EVs)	Phospholipid content (nM of lectin equivalents per 1x10^10^ EVs)	Carbohydrate content (μg per 1x10^10^ EVs)	Average size (nm)*
EVs Ca 3147	2.51 ± 0.56	1.47 ± 0.04	2.57 ± 0.90	168.2 ± 1.7
EVs Ca SC5314	3.95 ± 1.00	0.54 ± 0.09	2.34 ± 0.41	154.3 ± 1.1

*The average sizes of EVs are presented in accordance to the research of Karkowska-Kuleta et al. (2023).

**Figure 1:**
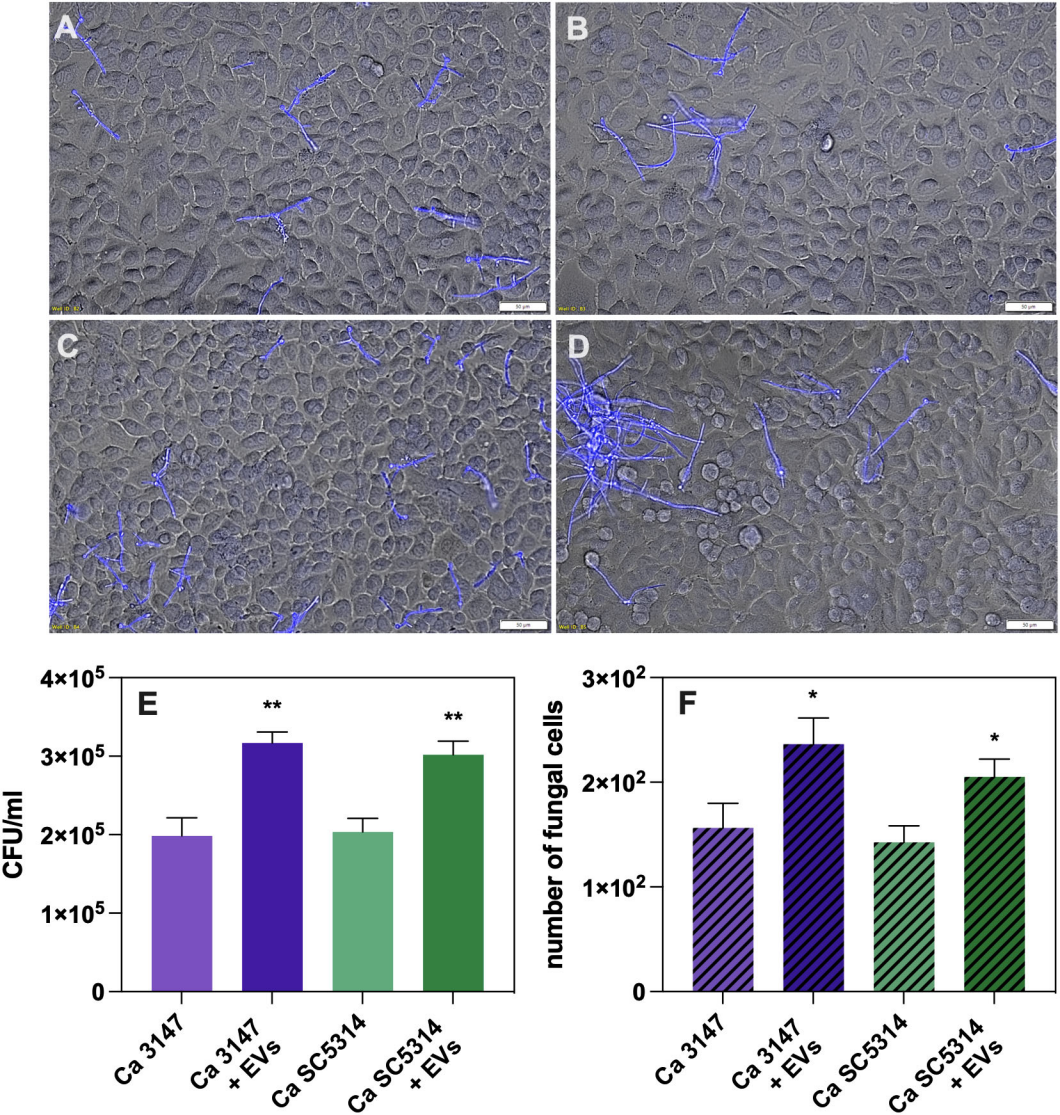
Adhesion to A549 epithelial cells of *C*. *albicans* strain 3147 **(A, C)** and SC5314 **(B, D)**, in the absence **(A, B)** or presence of *C*. *albicans* EVs **(C, D)**. The quantitative analysis of the number of fungal cells adhered to A549 cells determined with CFU counting **(E)** and microscopic counting of *C*. *albicans* cells in the field of view **(F)**. A representative result of the three independent experiments is presented. The statistical significance levels were marked with * for *p* < 0.05 and ** for *p* < 0.01.

**Figure 2:**
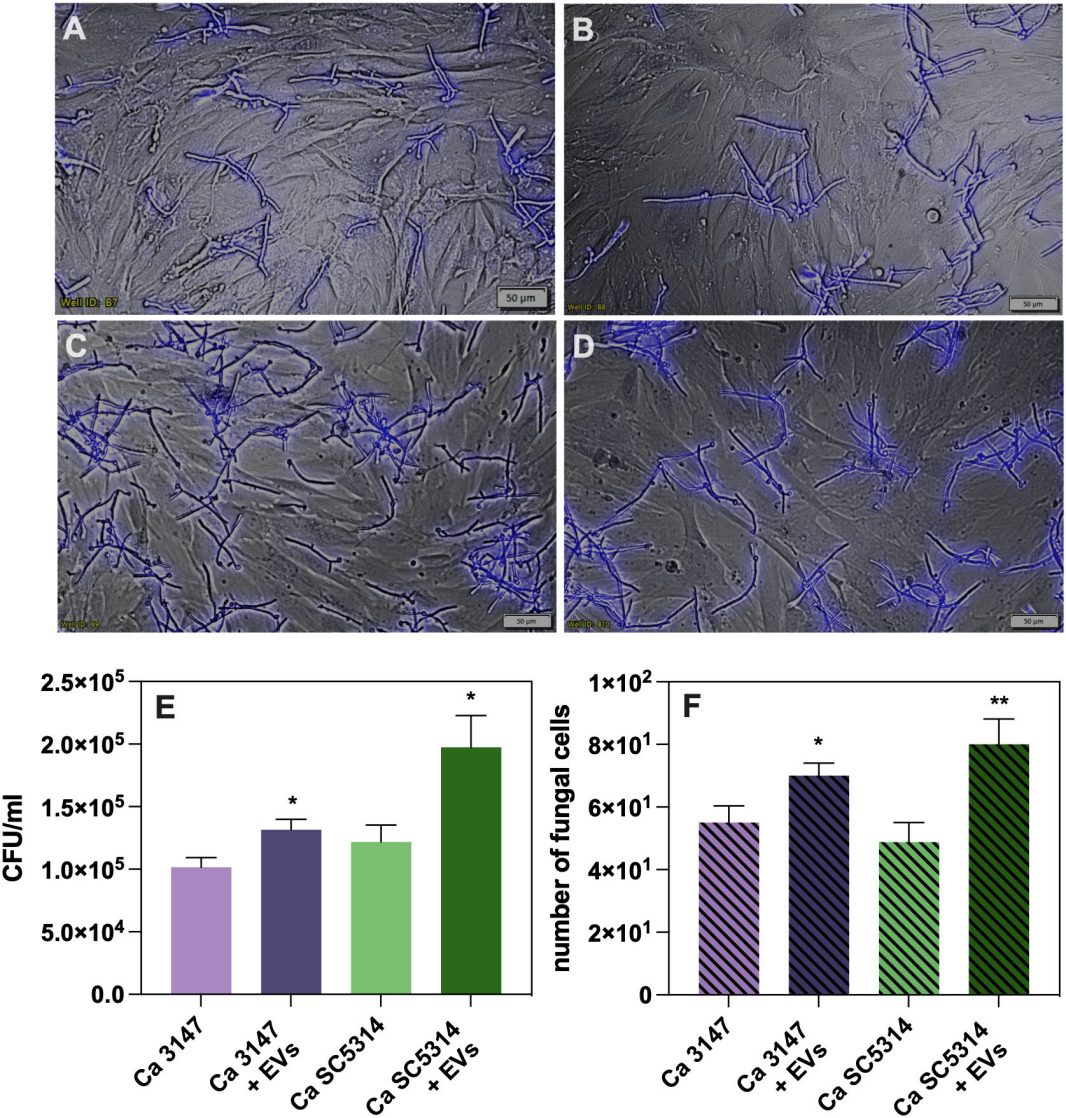
Adhesion to BJ fibroblast cells of *C*. *albicans* strain 3147 **(A, C)** and SC5314 **(B, D)**, in the absence **(A, B)** or presence of *C*. *albicans* EVs **(C, D)**. The quantitative analysis of the number of fungal cells adhered to BJ cell determined with CFU counting **(E)** and microscopic counting of *C*. *albicans* cells in the field of view **(F)**. A representative result of the three independent experiments is presented. The statistical significance levels were marked with * for *p* < 0.05 and ** for *p* < 0.01.

The functional properties of the isolated EVs were investigated after their characterization. First, the role of *C. albicans* EVs in promoting yeast cell adhesion to host cells was analyzed. A549 epithelial cells and BJ fibroblasts were used for the analysis and the microscopic examination and CFU counting were performed. After seeding epithelial or fibroblast cells in the wells of the microplate to form a monolayer, EVs from both *C. albicans* strains were added, along with yeast cells for further co-incubation. After this time, the fungal cell wall component – chitin – was stained with Calcofluor White. Microscopic observations revealed an increased number of yeast cells adhering to host cells in the presence of EVs compared to their absence (**Figure 1**). A similar trend was observed for fibroblast cells (**Figure 2**). To determine quantitative changes, CFU counting was performed and revealed statistically significant differences in promoting yeast adhesion to epithelial cells by EVs (**Figure 1E**). The increase in adhesion in the presence of EVs was comparable between the two strains of *C. albicans*. A similar trend was observed in adhesion of *C. albicans* cells to fibroblast cells, although the increase was not as evident (**Figure 2E**).

**Figure 3:**
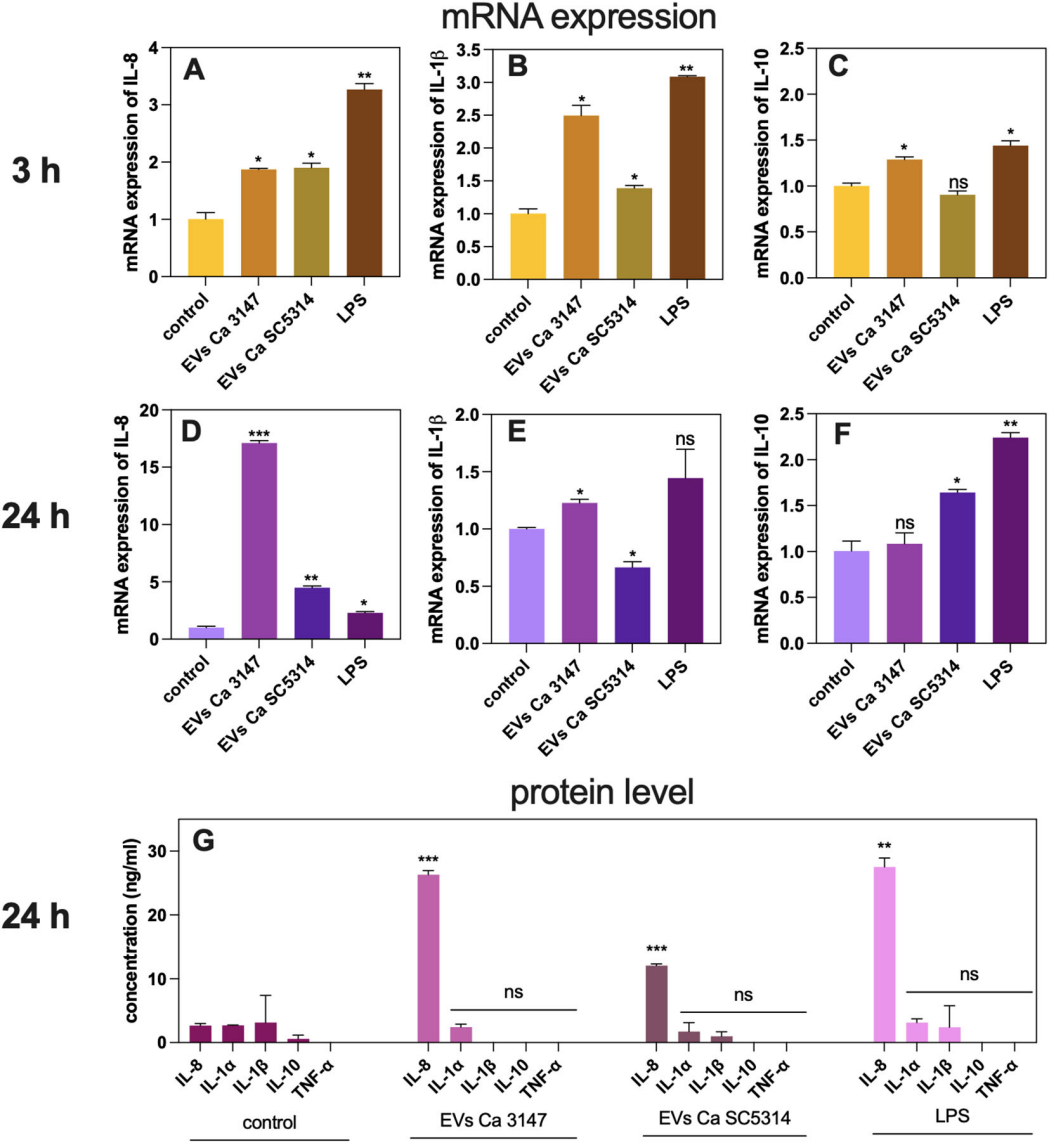
The analysis of the A549 epithelial cells response to *C. albicans* EVs tested on changes in gene expression and cytokine production. The mRNA gene expression of IL-8 after 3h **(A)** and 24h **(D)**, IL-1β after 3h **(B)** and 24h **(E)**, and IL-10 after 3h **(C)** and 24h **(F)**. Untreated cells served as a control. The analysis of cytokine production at the protein level **(G)**. LPS at concentration of 100 ng/ml was used as a positive control. The statistical significance levels were marked with ns for *p* 0.05, * for *p* < 0.05, ** for *p* < 0.01, and *** for *p* < 0.001.

After verifying the impact of EVs on the adhesion process, which represents the initial stage of *C. albicans* infection development, the subsequent response of A549 cells was investigated. The difference in mRNA expression of genes encoding cytokines IL-8, IL-1β, and IL-10 was assessed after 3 h and 24 h of incubating epithelial cells with EVs. Specifically, a 2-fold increase in the gene expression of IL-8 was observed after 3 h of incubation with EVs from both *C. albicans* strains (**Figure 3A**). After 24 h the effect was more pronounced, particularly for EVs of *C. albicans* strain 3147 which showed 17-fold increase, and for EVs of *C. albicans* strain SC5314, which demonstrated approximately 5-fold increase, both statistically significant (**Figure 3D**). The results for other genes encoding cytokines were less evident. The expression of the IL-1β gene increased by 2.5-fold after incubating A549 cells for 3 h with EVs derived from *C. albicans* strain 3147, while for EVs derived from *C. albicans* strain SC5314 the increase was only 1.4-fold (**Figure 3B**). After 24 h, the expression of this gene decreased 1.2-fold for EVs of *C. albicans* strain 3147 and 0.6-fold for those from strain SC5314, respectively (**Figure 3E**). For the gene encoding IL-10, a slight, but statistically significant upregulation, was observed after 3 h of incubation with EVs from *C. albicans* strain 3147, whereas a 1.5-fold increase was noticed after 24 h with EVs of *C. albicans* strain SC5314 (**Figures 3C, F**). A statistically significant increase in the IL-8 production at the protein level was observed using ELISA assay for both types of EVs, compared to untreated cells after 24 h; however, the effect of EVs from *C. albicans* strain 3147 was almost twice as great as that of EVs from *C. albicans* strain SC5314 (**Figure 3G**). The production of the other cytokines (IL-1α, IL-1β, TNF-α, IL-10) was also assessed; however, their levels were undetectable (**Figure 3G**).

**Figure 4:**
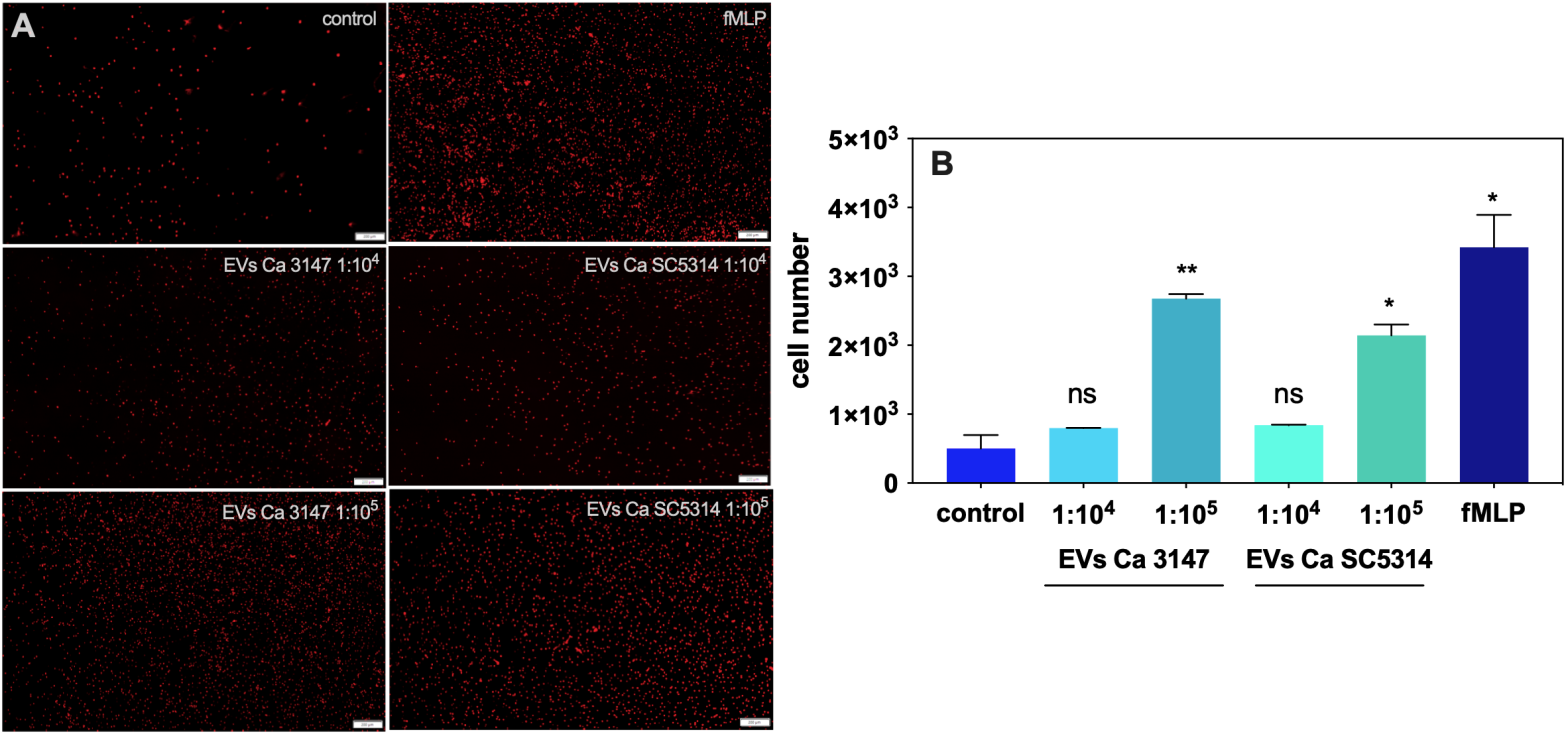
The chemotaxis of neutrophils in the presence of *C*. *albicans* EVs. **(A)** Microscopic imaging. **(B)** Counting of neutrophil cells. Untreated neutrophils served as a control. The fMLP at concentration of 10 µM was used as a positive control. The statistical significance levels were marked with * for *p* < 0.05, and ** for *p* < 0.01.

A further step in the host response to the presence of fungal particles are the neutrophil migration and activation, therefore a chemotaxis assay was performed with the use of fungal EVs. **Figure 4A** shows representative microscopic images of neutrophils, that migrated to the lower compartment of the chamber used for the chemotaxis assay. The quantitative analysis demonstrated an increased number of cells migrating to the medium containing EVs derived from both *C. albicans* strains (**Figure 4B**). The EVs from *C. albicans* strain 3147 were shown to be more potent chemoattractants for neutrophils than those from strain SC5314. However, neither phagocytosis of fungal EVs by neutrophils, nor a significant increase in the release of neutrophil extracellular traps (NETs) in the presence of these structures was observed (data not shown).

**Figure 5:**
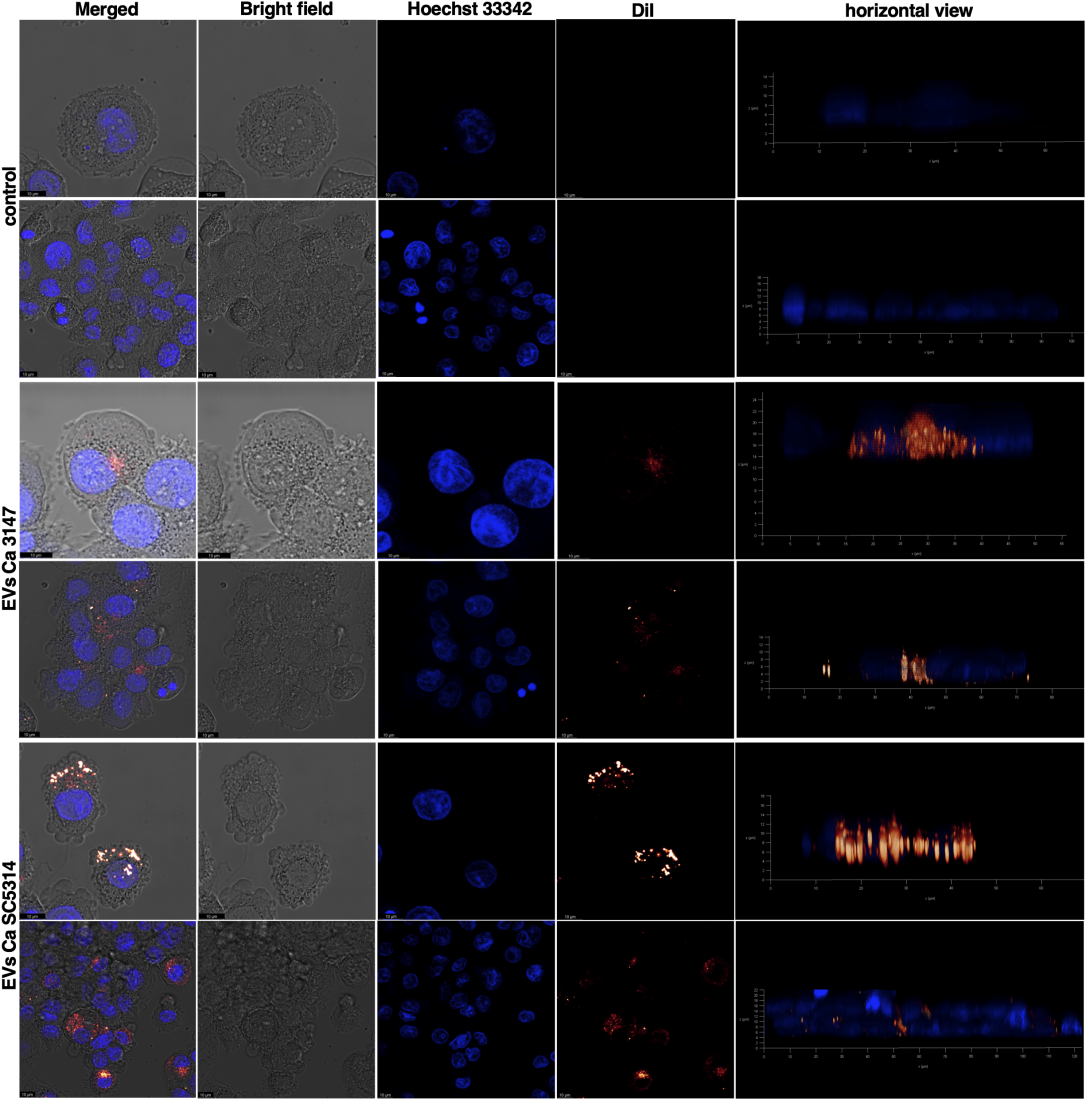
Uptake of *C. albicans* EVs labeled with DiI dye by THP-1 cells differentiated into macrophage-like cells after 24 h of incubation. The control is presented as untreated THP-1 cells incubated with fractions collected after a mock chromatographic separation of DiI dye used for EVs staining. The cell nuclei were stained with Hoechst 33342. As representative results the images showing the plane of the cell nucleus are presented. Visualization was performed using a Leica Stellaris confocal microscope. Scale bar: 10 μm.

**Figure 6:**
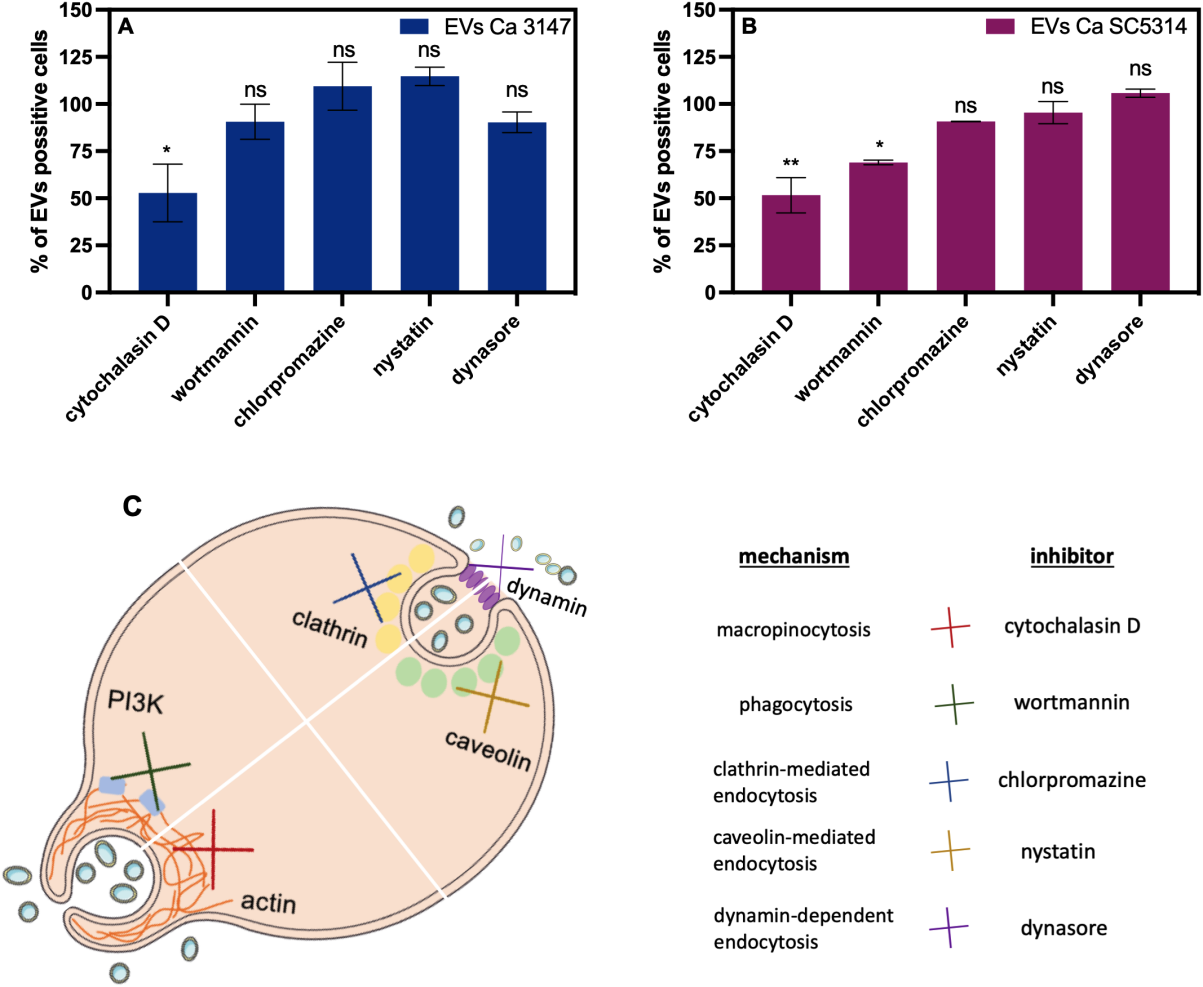
The mechanism of internalization of *C*. *albicans* EVs by THP-1 cells differentiated into macrophage-like cells. The flow cytometry analysis **(A, B)** was conducted after 6 h of incubation of THP-1 cells with EVs after treatment of cells with inhibitors. The control of the uptake of EVs by THP-1 cells untreated with any inhibitor was established as 100%. The average values from two independent experiments are presented with the statistical significance levels marked as ns for *p* 0.05, * for *p* < 0.05, and ** for *p* < 0.01 compared to the control. The mechanism of action of the used inhibitors is depicted **(C)**.

In the subsequent analysis, the internalization of fungal EVs by macrophage-like cells derived from THP-1 monocytes was examined and the use of EVs labelled with DiI lipophilic membrane stain facilitated analysis with confocal microscopy and flow cytometry. Cells incubated with a sample after the mock chromatographic separation of the Vybrant DiI Cell-Labelling solution were prepared as a control. The internalization of EVs by THP-1 cells has been confirmed for EVs of both *C. albicans* strains (**Figure 5** and **Supplementary Videos 1-4** in **Supplementary Material**). Moreover, for the first time the mechanism of *C. albicans* EVs uptake by human macrophage-like cells was analyzed. Host cells were independently treated with five different inhibitors blocking distinct internalization pathways (**Figure 6C**), and then incubated with fluorescently labelled EVs for 6 h. Flow cytometry analysis revealed a decrease in the number of cells that internalized both types of EVs by approximately 50% after treatment with cytochalasin D (which blocks macropinocytosis by inhibiting actin polymerization), compared to cells with preserved complete internalization capability as not treated with any inhibitor (**Figures 6A, B**). The pre-incubation of THP-1 cells with wortmannin, the inhibitor of phosphoinositide 3-kinase activity that blocks the internalization of EVs by phagocytosis, resulted in no changes in EVs uptake for vesicles produced by *C. albicans* strain 3147. However, a statistically significant reduction in EV-positive cells, to approximately 70% was observed for EVs from *C. albicans* SC5314 after wortmannin treatment (**Figures 6A, B**). For both types of *C. albicans* EVs, no changes in their internalization level by THP-1 cells were observed in cells pre-treated with chlorpromazine and nystatin, that inhibit internalization via clathrin- or caveolin-mediated endocytosis, respectively. In addition, also the treatment of cells with dynasore, which was re-supplemented during incubation with EVs due to readily reversible mechanism of dynamin activity inhibition, did not result in a significant reduction in EVs internalization levels in applied experimental approach (**Figures 6A, B**).

**Figure 7:**
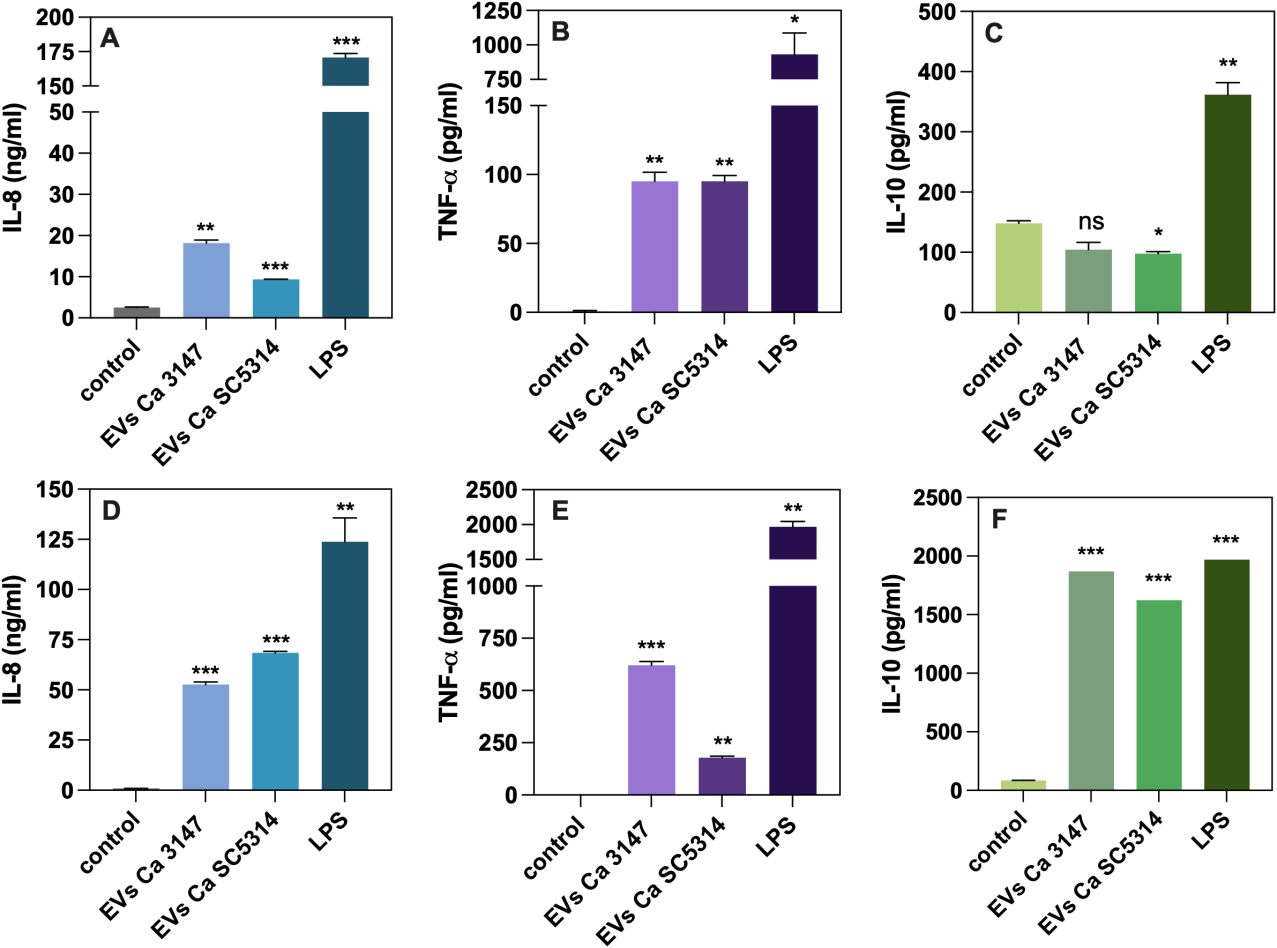
The analysis of cytokine production by THP-1 cells differentiated into macrophage-like cells **(A–C)** and blood-derived monocytes differentiated into macrophages **(D–F)**. The levels of IL-8 **(A, D)**, TNF-α **(B, E)** and IL-10 **(C, F)** are shown as representative results from three independent experiments. Untreated cells served as a control. LPS at concentration of 100 ng/ml was used as a positive control. The statistical significance levels were denoted as ns for *p* 0.05, * for *p* < 0.05, ** for *p* < 0.01, and *** for *p* < 0.001 in comparison to the control.

Further analysis focused on the cytokine release by human macrophage-like cells. Following incubation of THP-1 cells for 24 h with EVs derived from *C. albicans* strains 3147 and SC5314, the level of the proinflammatory and anti-inflammatory cytokines TNF-α and IL-10, respectively, and the chemokine IL-8, were assessed in the collected supernatants (**Figures 7A–C**). The obtained results demonstrated an increase in the production of IL-8 and TNF-α after exposure to EVs compared to untreated cells. Specifically, EVs from *C. albicans* strain 3147 had a greater impact on the release of IL-8 compared to the potential of EVs from strain SC5314 (**Figure 7A**). The level of TNF-α produced by THP-1 cells after exposition to EVs was comparable for both types of EVs (**Figure 7B**). Conversely, the release of IL-10 showed the opposite trend; the presence of fungal EVs resulted in lower levels of IL-10 compared to the control cells, with similar effects observed for EVs from both *C. albicans* strains (**Figure 7C**).

Further analysis focused on immune cells isolated from human whole-blood samples. After isolation, blood-derived monocytes were differentiated into macrophages and then exposed to fungal EVs from both strains of *C. albicans* for 24 h, similarly to the treatment of THP-1 cells. The levels of the same cytokines as those assessed for THP-1 cells were measured in the collected supernatants (**Figures 7D–F**). The performed analysis demonstrated an increase in the levels of IL-8 and TNF-α compared to untreated cells, but with a different pattern than that observed in the cell line model. Specifically, the level of IL-8 was higher after stimulation with EVs from strain SC5314 than with EVs from strain 3147 (**Figure 7D**). The production of TNF-α was significantly higher after exposure to EVs from strain 3147 than those from strain SC5314 (**Figure 7E**). In contrast to the response observed for THP-1 cells, the response of the blood monocyte-derived macrophages to fungal EVs resulted in releasing of IL-10 was markedly different. A significant increase in the production of this cytokine was observed in response to EVs from both strains of *C. albicans*, with comparable measured values (**Figure 7F**).

**Figure 8:**
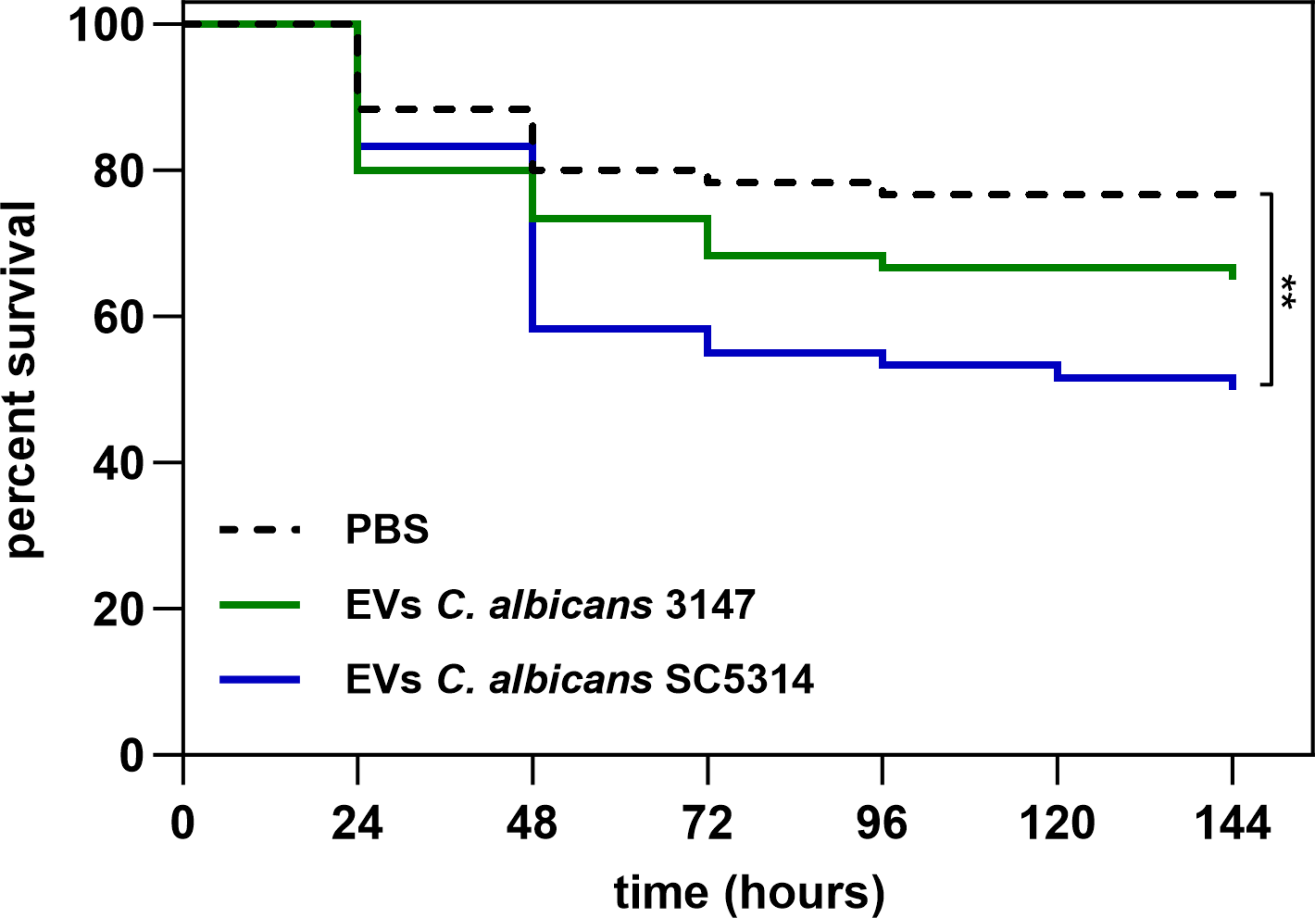
The survival of *Galleria mellonella* larvae after administration of 1 × 10^8^ of *C. albicans* EVs. PBS injection was used as a control. The statistical significance level was denoted as ** for *p* < 0.01.

To test *in vivo* the host response to EVs released by *C. albicans* cells forming biofilm structure, *Galleria mellonella* alternative model was applied. The effect of EVs on the survival of *G. mellonella* larvae was analyzed by exposing them to 1 × 10^8^ EVs per individual. The survival rate of larvae after the injection of EVs from strain 3147 was slightly lower to that of larvae injected with PBS, albeit statistically not significant. However, for EVs from strain SC5314 a significant increase in mortality was observed 2 days post-administration (**Figure 8**). After 4 days, and until the end of the experiment, about 70% of the larvae treated with EVs from strain 3147 survived, while about 50% of larvae treated with EVs from strain SC5314 exhibited mortality.

## 4 Discussion

EVs are structures with a lipid bilayer and of nanometer size produced by all types of prokaryotic and eukaryotic cells, containing various molecules as within their cargo. They play a key role in cellular communication and modulation of different physiological and pathological processes. The production of EVs by *C. albicans* fungi is well-documented in numerous studies. Diameters of *C. albicans* EVs vary slightly depending on the morphological form of the cells releasing EVs and the different culture conditions. Previously, we reported that biofilm-derived EVs produced by *C. albicans* strains 3147 and SC5314 are structures surrounded with lipid bilayer, with spherical shape and the sizes of the largest population of EVs ranged from 90 to 260 nm (Karkowska-Kuleta et al., 2023). The composition of EVs released by these two strains of *C. albicans* differs in terms of concentration of proteins and lipids, while carbohydrate amount was comparable. Higher concentration of lipids was observed for EVs released by *C. albicans* strain 3147 with simultaneously lower concentration of proteins. The inverse ratio of protein and lipid content was indicated for EVs from *C. albicans* strain SC5314.

EVs released by pathogens into the extracellular environment play an important functional role in multiple processes associated with the development of infection caused by these microorganisms. As presented in this study, one notable process is their involvement in communication between host and fungal cells. From the onset of fungal diseases, the presence of EVs derived from *Candida* yeasts proves to be significant. In the initial stage of colonizing host tissues, we demonstrated that EVs produced by both strains of *C. albicans* enhance the ability of yeasts to adhere to epithelial cells and fibroblast cells. Previously, a significant impact on the increased adherence of fungal cells to epithelium was observed for EVs released by *C. auris* in contact with HeLa cells, whereas EVs of *C. albicans* were shown not to be involved in this process (Zamith-Miranda et al., 2021). However, differences in the conditions applying pre-incubation or co-incubation with EVs, as well as the time of incubation, could be critical. In the studies involving HeLa cells, pre-incubation with yeast EVs lasted 1h before the whole cells were added, whereas our experiments involved a 3 h co-incubation of EVs and fungal cells added simultaneously. Previously, a parallel analysis of the impact of fungal EVs on the adherence of *C. albicans* to fibroblast cells had not been performed.

In the further stages of the progression of candidiasis, the ability to release EVs by *C. albicans* is also significant. The presence of fungal EVs can modulate the host immune responses by multiply mechanisms engaging, among others, neutrophils or macrophages. The important antifungal mechanisms used by neutrophils are the generation of ROS, release of NETs, or the activation of phagocytosis (Kenno et al., 2016; He et al., 2022; Zawrotniak et al., 2023). In our research, *C. albicans* EVs derived from both examined strains of *C. albicans* were identified as active chemoattractants for blood-derived neutrophils. Surprisingly, no activation of either phagocytosis, or NETs release was observed, although several virulence factors—proven to be components of EVs, such as proteins, nucleic acids, and lipids—have been identified as involved in generating such responses. The examples of these molecules include Saps (secreted aspartic proteases), mannans and glucans - the components of *Candida* cell wall, Als3 – the main adhesin, and Eno1 – the enzyme representing the moonlighting proteins (Zawrotniak et al., 2017). Moreover, the extracellular nucleic acids – another component of EVs cargo and compound structuring biofilm matrix, was shown to be the chemotactic factor, inducing also ROS production and triggering NETosis (Smolarz et al., 2021). Nevertheless, the participation of *C. albicans* EVs, as one of the virulence factors, in the response of neutrophils to fungal infection had not been investigated previously.

In our recent work we demonstrated the ability of THP-1 macrophage-like cells to internalize EVs derived from the yeast-like cells of *C. albicans* strain 3147 cultured on the solid YPD medium, however, the mechanisms of this process have not been investigated in detail (Kulig et al., 2023). In this work we demonstrated the internalization of biofilm-derived EVs released by both tested *C. albicans* strains after their incubation with THP-1 cells. With the use of specific inhibitors of selected uptake pathways, we considered for the first time the most probable mechanism exploited by THP-1 cells for the internalization of *C. albicans* EVs. The obtained results indicated that macropinocytosis was identified as one of the mechanisms involved in the uptake of EVs produced by biofilms of both *C. albicans* strains. Nevertheless, for EVs from strain SC5314, phagocytosis was additional mechanism identified herein as involved in their uptake by THP-1 macrophage-like cells. Moreover, under the conditions tested, the EVs internalization process did not seem to involve clathrin- or caveolin-mediated endocytosis. Under the applied experimental conditions, it seems also that the internalization process did not depend on dynamin activity. However, it is important to notice the ease of reversibility of the dynasore inhibitor action. Nonetheless, as it was previously demonstrated after removing of dynasore from cell culture during washing steps, the inhibitory effect was preserved for 20 minutes, and after inhibitor re-supplementation, the endocytosis inhibition was reconstituted within few minutes (Macia et al., 2006). Due to the characteristic of the action of dynasore, the application of further various experimental conditions could allow to obtain more comprehensive description of the effect of dynamin-related mechanism in the process of EVs internalization. For comparison, the rapid internalization of EVs from *Cryptococcus gattii* by J774 macrophages was also determined as an active uptake involving macropinocytosis, where the mechanism was examined using actin polymerisation inhibitors such as cytochalasin D and latrunculin A, or process depending on lipid rafts (Bielska et al., 2018; Sabatke et al., 2023). Furthermore, in the studies describing internalization of EVs produced by cells of *C. albicans* strain 11 by murine macrophages and dendritic cells, an uptake pathway based on the involvement of lipid rafts has been also suggested (Vargas et al., 2015). Whereas the analysis performed by Brown Harding et al. (2024) for EVs from *C. albicans* strain SN250 with the use of murine cells showed increased presence of viperin after internalization of fungal vesicles. Pre-treatment with dynasore before adding fungal EVs resulted in no viperin release, indicating that dynamin-dependent endocytosis was the main mechanism of EVs internalization by murine macrophages (Brown Harding et al., 2024). Therefore, according to literature reports, different uptake pathways may be involved depending on the cell type, the heterogeneity of the EVs population or the specific conditions influencing the presence of different molecules on the surface of both interacting partners (Mulcahy et al., 2014; French et al., 2017). This multifactorial impact on the process of EVs internalization suggest the need of application of complex experimental approach in further research.

The immunomodulatory potential of *C. albicans* EVs on THP-1 cells was also tested. The analysis of cytokine secretion revealed the increased production of IL-8 and TNF-α after 24 h incubation of THP-1 cells with both types of *C. albicans* EVs, while the level of IL-10 was decreasing in comparison to untreated cells. In the research by Martinez-López et al. (2022), EVs obtained from *C. albicans* strains SC5314, grown in the selected culture media to promote yeast or hyphal morphology, induced different responses of the host cells. Due to various culture conditions, the cargo of released EVs differed and the cytotoxicity increased with larger amount of virulence-related proteins for vesicles produced by hyphal cells. The changes in the production of cytokines IL-10 and IL-12 by host cells were not significant after 24 h, while the level of TNF-α was not detected after stimulation with EVs released by yeast cells but slightly increased after contact with EVs produced by hyphal cells (Martinez-López et al., 2022). The differences between our results and those presented by Martinez-López et al. (2022) may arise from varying culture conditions, indicating that environmental changes can alter the properties of *C. albicans* EVs. On the other hand, the response of blood-derived monocytes differentiated into macrophages revealed dissimilarity in the production of IL-10 when compared with the results obtained for THP-1 cells. The level of all three tested cytokines was increased after stimulation of macrophages with both types of *C. albicans* EVs. For comparison, the response of RAW 264.7 macrophage cells, and bone marrow-derived macrophages (BMDM) to contact with EVs released by clinically isolated *C. albicans* strain 11 resulted in higher levels of TNF-α, IL-10, IL-12p40 and TGF-β cytokines (Vargas et al., 2015).

The immune response induced by EVs also varies according to other species of *Candida* genus. For EVs produced by biofilms of non-*albicans Candida* species — *C. parapsilosis, C. tropicalis* and *N. glabratus* — the response of THP-1 macrophage-like cells tends to be predominantly proinflammatory, with increased levels of TNF-α after 24 h stimulation, especially for *N. glabratus* EVs. On the other hand, the production of IL-10, an anti-inflammatory cytokine, decreased in comparison to untreated cells, with the most significant effect observed for EVs from *C. tropicalis*. The release of IL-8 was the highest following contact with *N. glabratus* EVs, slightly lower for *C. parapsilosis* EVs, and with the lowest effect observed after stimulation with *C. tropicalis* EVs (Kulig et al., 2022).

Furthermore, the host environment, such as the concentration of carbon dioxide, and oxidative stress generated during infection, alter the conditions within the host organism, influencing the responses of microorganisms that cause infections. EVs released by *C. albicans* under these conditions exhibited a proinflammatory effects, demonstrated by an increase in TNF-α level, particularly in response to *C. albicans* EVs produced under oxidative stress conditions, and by a decrease in IL-10 level compared to untreated cells (Kulig et al., 2023).

The production of IL-8 was also higher when stimulated with EVs obtained from yeasts grown under oxidative stress conditions (Kulig et al., 2023). The observed effects on host response might result from changes in the lipid profile similarly as for EVs released by *C. albicans* strain ATCC 64548 in the presence of menadione, a factor that induces oxidative stress conditions (Trentin et al., 2023).

In *G. mellonella* model, EVs from the *C. albicans* strain SC5314 significantly influenced the mortality rate of the larvae, while EVs derived from strain 3147 did not demonstrate negative impact on the survival rate of *G. mellonella*. The effect of the other strain of *C. albicans* EVs on the model organisms showed that the pretreatment with *C. albicans* EVs before the injection of whole fungal cells resulted with higher survival rate of *G. mellonella* (Vargas et al., 2015), indicating protective role of the immunization with vesicles. Furthermore, EVs administered to mice before inducing candidiasis influenced higher levels of TNF-α, IL-4, IL-10, IL-12p70, TGF-β and IFN-γ (Vargas et al., 2020). The analysis of the protein cargo of EVs from *C. albicans* SC5314 reveal the presence of candidalysin or its precursor, mainly in EVs released by hyphal and biofilm-forming fungal cells (Zarnowski et al., 2018; Martinez-López et al., 2022; Karkowska-Kuleta et al., 2023). However, this peptide was not detected in the EVs of *C. albicans* strain 3147. Candidalysin is a peptide responsible for activating acute signaling pathways and immune responses, and is also known as factor inducing damage of host epithelium and hemolysis (Moyes et al., 2016; Richardson et al., 2018, 2022; Swidergall et al., 2019; Mogavero et al., 2022). The treatment with this peptide has been demonstrated to decrease the survival rate in model organisms, including zebrafish and mice (Swidergall et al., 2019). The presence of candidalysin in the EVs from the examined *C. albicans* strain may also be linked to the higher mortality rate observed in the *G. mellonella* model used in this study, following the administration of EVs.

**Figure 9:**
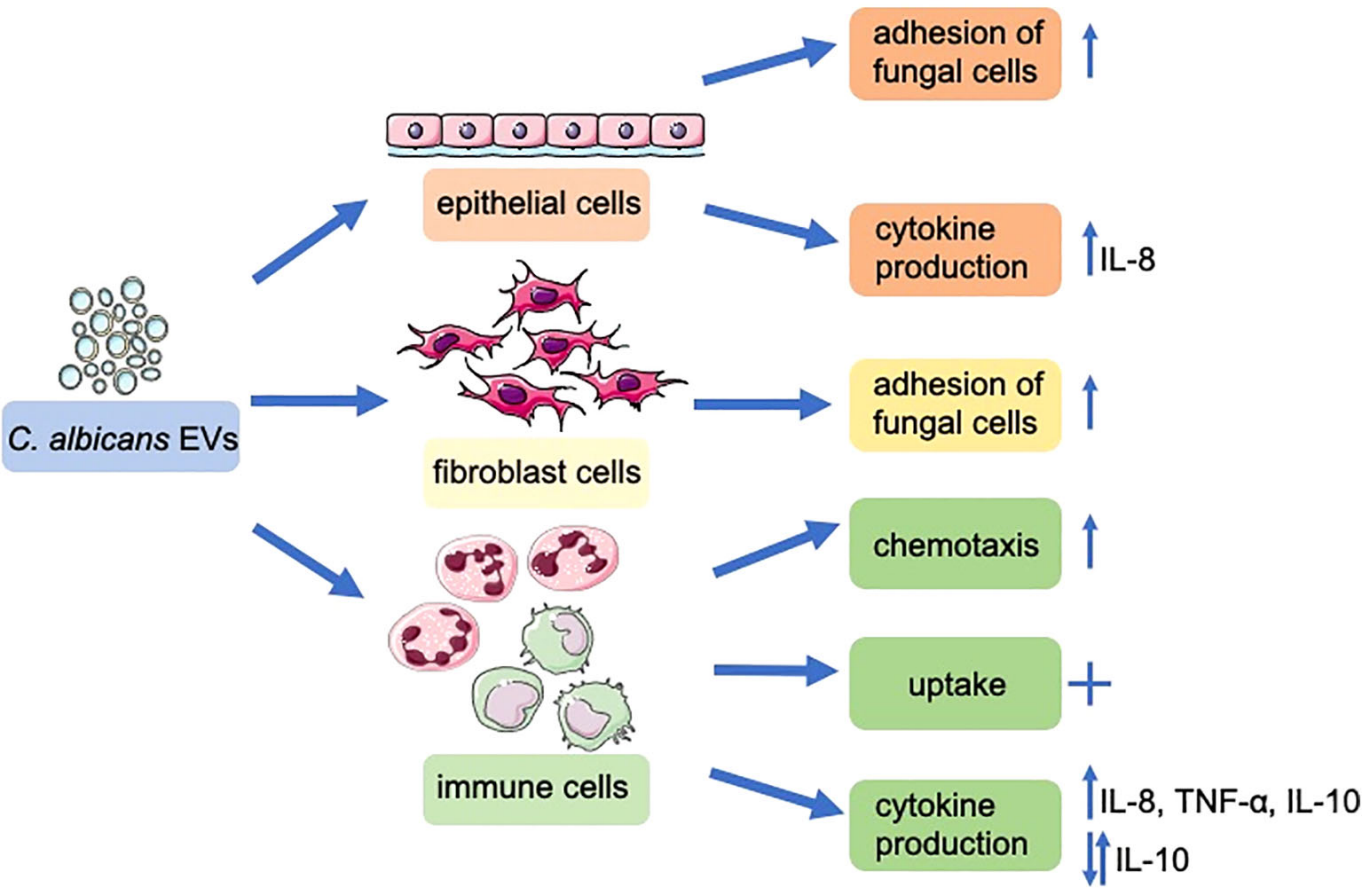
The functional properties of *C. albicans* strain 3147 and SC5314 EVs derived by biofilm forming cells on host cells. The figure was partly generated using Servier Medical Art, provided by Servier, licensed under a Creative Commons Attribution 3.0 unported license.

The involvement of *C. albicans* EVs in the host-pathogen interactions, as demonstrated in this study, highlights the contribution of epithelial and fibroblast cells, along with immune cells such as neutrophils and macrophages, in response to these structures (schematically presented in **Figure 9**). This emphasizes the crucial role of EVs released by *C. albicans* cells forming biofilm structure in eliciting host responses.

## Data Availability

The raw data supporting the conclusions of this article will be made available by the authors, without undue reservation.

## Supplementary material

Supplementary Video 1 Visualization of *C. albicans* strain 3147 EVs internalization by THP-1 cells.

Supplementary Video 2 Visualization of *C. albicans* strain 3147 EVs internalization by THP-1 cells in the wide field of view.

Supplementary Video 3 Visualization of *C. albicans* strain SC5314 EVs internalization by THP-1 cells.

Supplementary Video 4 Visualization of *C. albicans* strain SC5314 EVs internalization by THP-1 cells in the wide field of view.
